# A Beam Steering Vector Tracking GNSS Software-Defined Receiver for Robust Positioning

**DOI:** 10.3390/s25061951

**Published:** 2025-03-20

**Authors:** Scott Burchfield, Charles Givhan, Scott Martin

**Affiliations:** 1 Integrated Solutions for Systems (IS4S), Auburn, AL 36830, USA; scott.burchfield@is4s.com; 2 Mechanical Engineering Department, Auburn University, Auburn, AL 36849, USA; smm0008@auburn.edu

**Keywords:** GNSS, vector tracking, antenna arrays, robust navigation, software-defined radios

## Abstract

Global navigation satellite systems are the best means of navigation for dynamic platforms. However, interference, line-of-sight blockages, and multipath are destructive to receiver operations. Advanced receiver architectures like vector tracking loops have been shown to be more resilient in tracking during degraded signal environments and dynamic scenarios. Additionally, controlled reception pattern antennas can be used to steer the effective antenna gain pattern to resist interference. This work introduces algorithms for a software-defined radio that combines vector tracking loops with a phased antenna array to digitally steer beams for the amplification of signals of interest so that the effects of signal degradation and multipath can be reduced. The proposed receiver design was tested on dynamic live sky data in multipath-rich environments and compared against traditional scalar receivers with and without beamforming as well as robust commercial receivers. The results showed that beam steering receivers were obtaining the expected amplification and that the vector tracking with beam steering was able to provide better positioning and signal tracking performance than the other implemented software receivers and provide continuous measurements where the commercial receiver failed to track degraded signals.

## 1. Introduction

The use of a Global Navigation Satellite System (GNSS) is the best method for obtaining absolute position and timing information of a dynamic platform. However, GNSSs are inherently vulnerable to interference and disruptions, both intentional and environmental, due to low broadcast signal power, large propagation distances, and reflections of the signal. As a result, it is imperative that GNSS receivers have a robust design such that they can operate continuously despite environmental degradations of the incoming signal. Therefore, it is necessary for investigations into adaptations and algorithms that can toughen the GNSS receiver to enable it to resiliently track signals through increasingly degraded conditions. This work focuses on adaptations to the receiver algorithms and antenna system to provide improvement in tracking of GNSS signals in degraded and multipath environments.

For GNSS navigation, the most vulnerable part of the receiver is the tracking of the individual received channels. While improving filter design can allow for more robust signal tracking, one of the best ways to improve GNSS signal processing is to treat the signal parameters as functions of the navigation states in a joint estimation design. The two most common algorithms for achieving this are vector tracking loops (VTL) and direct position estimation (DPE). Vector tracking applies the measurements of signal parameters from each channel directly as navigation measurements to a centralized navigation filter and determines future signal replicas from the navigation states. This allows VTLs to support degraded and low signal power channels with proper estimates of the local replica in situations where equivalent traditional or scalar tracking loops would fail [1,2,3,4]. It has also been shown that VTLs navigation state-based updates improve the effectiveness of handling higher-order dynamic stresses [5,6]. Research has also shown that VTLs have an inherent resilience to tracking in environments where non-line-of-sight (NLOS) or multipath signals are prevalent, as the VTL is less likely for a channel to lock on to an NLOS signal because it does not follow the navigation state estimate of signal parameters [7,8]. Direct Position Estimation also provides local replicas as a function of navigation states, but it performs a maximum likelihood estimation approach that correlates all signals over all possible combinations of candidate states to find the states with the maximum correlation. DPE enables receivers to provide navigation solutions in incredibly low power signal conditions, but it also guarantees the full presence of all NLOS signals in the correlation plane, which can skew navigation accuracy [9,10].

Robust algorithms have been consistently shown in the literature to improve receiver performance in degraded environments. Also, recent advances in applications of machine learning have been successfully leveraged for the detection and classification of multipath signals, which provides an additional layer of integrity for improved positioning [11,12]. However, these methods, while adding resistance, do nothing to aid in the mitigation of multipath signals. To mitigate multipath signals, changes to the physical hardware can be implemented, particularly the antenna system, which can provide spatial filtering. The use of antenna arrays allows for the manipulation of the gain pattern, which can amplify or attenuate signals based on look direction. These arrays, called controlled reception pattern arrays (CRPA), provide spatial degrees of freedom, which typically are used for filtering strong radio frequency interference (RFI) [13,14]. There also are new array designs such as time-modulated arrays (TMA) that provide additional possibilities in array pattern generation that can be adapted into use for radio navigation [15,16]. CRPAs, while primarily used in navigation as a means of mitigating RFI, can also be used to improve the detection of authentic signals while also providing some multipath resilience of GNSS through beam-forming techniques [17,18,19,20]. However, the study of the use of antenna arrays with GNSSs has been limited due to the United States export control laws, so there is a need for the study of performance gains available to using an antenna array in non-RFI degraded environments.

Beamforming techniques can be applied in conjunction with robust tracking algorithms to provide better detection thresholds and multipath resilience, which will provide better positioning performance in everyday degraded environments. In the literature, the German Aerospace Center (DLR) has published works on the implementation of a vector tracking receiver that is coupled with a beamforming array to maximize the received signal power and has demonstrated this system on dynamic platforms [21,22]. The DLR implementation uses a post-correlation beamformer where the directions of arrival (DOA) of the signals are determined for use in beam steering. The DLR work has a heavy focus on resilience to RFI environments in their work as opposed to a common multipath environment. The presence of multipath can distort correlations and bias carrier phases that could degrade DOA and therefore beamforming performance. In this paper, the authors provide algorithms for a beam steering vector tracking software-defined radio (SDR) that is experimentally validated in multipath environments. The purpose is to validate the benefit of using robust receiver design and beamforming to provide continual GNSS navigation in everyday use cases. This work uses a pre-correlation beamformer that takes attitude and ephemeris information into account to then digitally steer each channel in the navigation state-determined look direction of the emitter. The proposed receiver from this work will provide the following:

- An outline of a simple implementation of a beamforming VTL for robust performance in everyday degraded environments.
- An evaluation of a deterministic beamformer that uses a navigation state-determined DOA to prevent NLOS distortions from affecting signal-based DOA estimates.
- A pre-correlation beamformer that will require fewer correlations than in other post-correlated designs as in [21,22].
- A receiver architecture that has modularity with additional sensors for attitude information and deep integration.
- A validation of the proposed receiver in both simulation and in live-sky experiments with dynamic and degraded signal environments.

The rest of the work will be organized as follows. A presentation of the algorithms used in the receiver will be presented in Section 2.1. This will be followed by simulation results outlining the performance gains of using robust receiver architectures and antenna arrays in Section 2.2. Next, details of the dynamic experimental data collection will be provided in Section 3. Lastly, the results of the proposed beam-forming vector tracking receiver will be demonstrated on live sky dynamic data through heavy multipath conditions in Section 4, and discussion and conclusions will be in Section 5.

## 2. Algorithms and Simulation Studies

This section will present an overview of the algorithms used in the receiver and a simulation study of beam steering performance in degraded environments.

### 2.1. Overview of Algorithms

#### 2.1.1. An Overview of Vector Tracking

**Figure 1:**
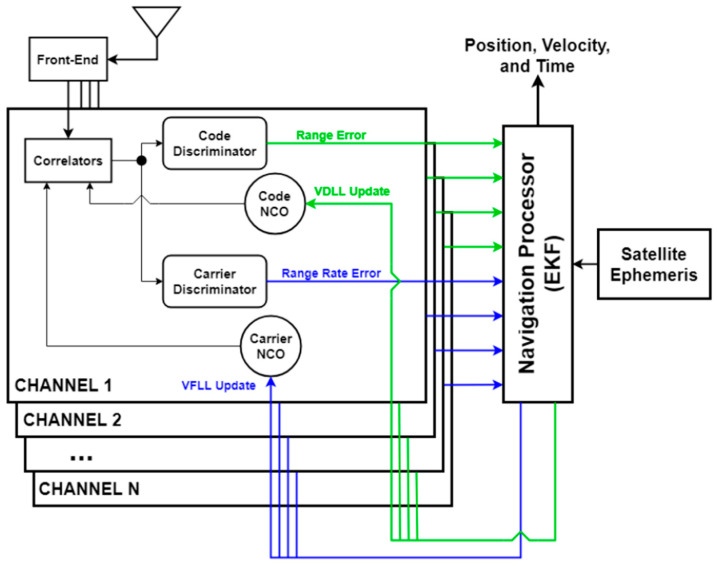
A diagram overviewing the architecture of a vector tracking loop in a GNSS receiver.

For a traditional or scalar tracking GNSS receiver, each channel generates a local replica from a numerically controlled oscillator (NCO) and correlates the replica with an incoming signal. The correlations are converted into measurements of replica parameter error called discriminators. The discriminator is passed through a filter and is then used to update the NCO for the next correlation period. Once the NCO estimates have converged and the satellite data are decoded, the NCOs are converted into measurements of range and range rate and are given to a centralized navigator to solve for the position velocity and timing (PVT) states. Vector tracking treats the local replicas directly as functions of the navigation states. Each channel has its NCOs determined by the current navigation state, and the discriminators are applied directly as measurements to the navigator rather than to isolated loop filters. Once the navigation state is updated by the channel, the navigator then updates the NCOs of that channel. A diagram of the process is presented in Figure 1.

#### 2.1.2. The Central Navigation Filter

The VTL receiver in the work uses an extended Kalman filter (EKF) with an eight-element state vector given as:

(1) $$x={\left[x,\dot{x},y,\;\dot{y},z,\dot{z},b,\dot{b}\right]}^{T}.$$

The states are the earth-centered earth-fixed (ECEF) Cartesian position coordinates, *x*, *y*, *z*, and their velocities, $\dot{x},\dot{y},\dot{z},$ as well as the receivers clock bias *b* in meters and drift $\dot{b}$ in meters per second. The filter uses a constant velocity model. The discretized process model is given by

(2) $$Q=\left[\begin{array}{cccc} Q_{p} & 0 & 0 & 0\\ 0 & Q_{p} & 0 & 0\\ 0 & 0 & Q_{p} & 0\\ 0 & 0 & 0 & Q_{c} \end{array}\right]$$

where the diagonal elements are

(3) $$Q_{p}=\sigma_{p}^{2}\left[\begin{array}{cc} \frac{{\delta t}^{3}}{3} & \frac{{\delta t}^{2}}{2}\\ \frac{{\delta t}^{2}}{2} & \delta t \end{array}\right]$$

and

(4) $$Q_{c}=\left[\begin{array}{cc} \frac{c^{2}h_{0}}{2}\delta t+\frac{2{\left(c\pi \right)}^{2}h_{-2}{\delta t}^{3}}{3} & \frac{2{\left(c\pi \right)}^{2}h_{-2}\delta t}{2}\\ \frac{2{\left(c\pi \right)}^{2}h_{-2}\delta t}{2} & 2{\left(c\pi \right)}^{2}h_{-2}\delta t \end{array}\right].$$

The acceleration variance, $\sigma_{P}^{2}$, is tunable based on expected dynamics. It is assumed that the acceleration variance is equal in all Cartesian directions. The process noise terms are all functions of δt which is the time since the last measurement was applied. In VTLs, the measurements are not available until the closure of an integration period between the incoming signal and the local replica from the NCO, where they must be immediately applied so that the updated state can provide the next NCO. This causes a series of asynchronous measurement updates on a channel-by-channel basis that requires a varying time in the receiver model. The clock process model from Equation (4) is based on Allan variance parameters, $h_{0}$ and $h_{-2}$, as well as the speed of light, *c*. Parameter values for a temperature-controlled crystal oscillator (TCXO) from [23] are used in this work. The measurement model for the VTL is provided in the next section.

#### 2.1.3. Vector Tracking Measurement Models

The standard measurement set for GNSSs is pseudorange and pseudorange rates from each channel. The measurements for each are given in Equations (5) and (6), respectively.

(5) $${\tilde{\rho }}_{j}=\left(t_{r}-t_{t}^{j}\right)*c\;$$

(6) $${\tilde{\dot{\rho }}}_{j}=-\lambda_{L1}*f_{D}^{j}$$

The pseudorange measurement for the $j^{th}$ satellite, ${\tilde{\rho }}_{j}$, is a function of $t_{r}$ the receiver reception time, and $t_{t}^{j}$, the channel’s transmission time. The transmission time is a function of the channel’s estimated received code phase. The pseudorange rate measurement, ${\tilde{\dot{\rho }}}_{j}$, is a function of $f_{D}^{j}$, the channel’s Doppler frequency, and $\lambda_{L1}$, the wavelength of the carrier frequency. This work uses only the GPS L1 frequency. The pseudorange measurement model for the $j^{th}$ satellite is given below as

(7) $$\hat{\rho_{j}}=\|x_{j}^{sv}-\hat{x}\|+\hat{b}+\eta_{R}$$

The pseudorange estimate, ${\hat{\rho }}_{j}$, is the norm of $x_{j}^{sv}={\left[x_{j}^{sv},\;y_{j}^{sv},\;z_{j}^{sv}\right]}^{T}$, the ECEF position of the $j^{th}$ satellite, and $\hat{x}={\left[\hat{x},\;\hat{y},\;\hat{z}\right]}^{T}$, the ECEF position estimates of the receiver plus the estimated receiver clock bias, $\hat{b}$. It is modeled as having an additive white gaussian noise (AWGN) term, $\eta_{R}$. The pseudorange rate model is the time derivative of Equation (7), and is shown below as

(8) $$\hat{{\dot{\rho }}_{j}}=\frac{{\left(x_{j}^{sv}-\hat{x}\right)}^{T}({\dot{x}}_{j}^{sv}-\hat{\dot{x}})}{\left\|x_{j}^{sv}-\hat{x}\right\|}+\hat{\dot{b}}+\eta_{RR}$$

The pseudorange rate measurement, $\hat{{\dot{\rho }}_{j}}$, is a function of $x_{j}^{sv}$, the satellite position, ${\dot{x}}_{j}^{sv}$, the satellite velocities, $\hat{x}$, the estimated receiver position, $\hat{\dot{x}}$, the estimated receiver velocities, and $\hat{\dot{b}}$, the estimated receiver clock drift. It is also modeled as having an AWGN term, $\eta_{RR}$. The geometry matrix that maps the receiver states to the observations is the Jacobian of Equations (7) and (8) and is shown below.

(9) $$\left[\begin{array}{c} {\tilde{\rho }}_{j}\\ \tilde{\dot{\rho }} \end{array}\right]=Hx=\left[\begin{array}{cccccccc} \frac{-\left(x_{j}^{sv}-\hat{x}\right)}{\left\|x_{j}^{sv}-\hat{x}\right\|} & 0 & \frac{-\left(y_{j}^{sv}-\hat{y}\right)}{\left\|x_{j}^{sv}-\hat{x}\right\|} & 0 & \frac{-\left(z_{j}^{sv}-\hat{z}\right)}{\left\|x_{j}^{sv}-\hat{x}\right\|} & 0 & 1 & 0\\ 0 & \frac{-\left(x_{j}^{sv}-\hat{x}\right)}{\left\|x_{j}^{sv}-\hat{x}\right\|} & 0 & \frac{-\left(y_{j}^{sv}-\hat{y}\right)}{\left\|x_{j}^{sv}-\hat{x}\right\|} & 0 & \frac{-\left(z_{j}^{sv}-\hat{z}\right)}{\left\|x_{j}^{sv}-\hat{x}\right\|} & 0 & 1 \end{array}\right]x$$

In a traditional receiver, the individual channel loop filters would refine the estimates of code phase and Doppler to provide the measurements of pseudorange and pseudorange rate to the navigation filter. In the vector tracking receiver, since the initial position estimate is known, the residual between the measurements and the measurement estimates is assumed to be the measured tracking errors from the discriminators on each channel. The discriminators are then applied directly from the channels to the navigation filter. The vector design used in this work is a vector delay frequency lock loop (VDFLL). The receiver uses the traditional six correlators: in-phase early, in-phase prompt, in-phase late, quadrature early, quadrature prompt, and quadrature late (IE, IP, IL, QE, QP, and QL). These correlators are converted into errors of code phase and carrier frequency by discriminator functions. The code discriminator used in this work is

(10) $$\theta_{DLL}=\frac{1}{2}\frac{\sqrt{IE^{2}+QE^{2}}-\sqrt{IL^{2}+QL^{2}}}{\sqrt{IE^{2}+QE^{2}}+\sqrt{IL^{2}+QL^{2}}},$$

where $\theta_{DLL}$ is the normalized early-minus-late correlator power discriminator using one chip early to late correlator spacing. The frequency discriminator in this work is

(11) $$\theta_{FLL}=\frac{atan2\left(IP_{1}*QP_{2}-IP_{2}*QP_{1},IP_{1}*IP_{2}+QP_{1}*QP_{2}\right)}{2\pi \left(\frac{T}{2}\right)},$$

where $\theta_{FLL}$ is the cross-dot frequency discriminator using the prompt correlators from the first and second half of the integration period, *T.* The integration period used is 20 milliseconds, as limited by the data rate of GPS L1 C/A. With the discriminators defined, the pseudorange and pseudorange rate residuals provided to the navigator for the VDFLL are given as

(12) $$\delta \rho =\theta_{DLL}*\lambda_{CA}+\left(\tilde{\rho }-\hat{\rho }\right)$$

(13) $$\delta \dot{\rho }=-\theta_{FLL}*\lambda_{L1}+\left(\dot{\tilde{\rho }}-\dot{\hat{\rho }}\right),$$

where the discriminators are scaled by, $\lambda_{CA}$, the wavelength of the code, and $\lambda_{L1}$, the wavelength of the carrier, respectively, to make them units of range and range rate. The vector residuals in Equations (12) and (13) also contain the traditional scalar measurement residuals from Equations (5)–(8). The continued inclusion of the traditional range and range rate residual is due to the asynchronous nature of VTLs. The correlation process between the local replica and the incoming signals is aligned to the data bit of every channel. After the correlation has been completed, the measurements must then be immediately applied to the navigation filter to update the position states so that the NCOs can be updated for the next correlation period. This prescribed time of application of every measurement causes the asynchronous updates, which means that the navigation states that were used to create the NCOs for the measurements being applied have since been updated by every other channel during the correlation period. Therefore, the discriminators available do not fully represent the residual due to the change in state. While there do exist solutions involving back propagation of the filter and additional states such as shown in [24], a simpler and effective way to account for the change in state is to include the traditional residual.

The measurement models treated both pseudorange and pseudorange rate as having AWGN terms. Both those terms are modeled as zero mean with variances such that $\eta_{R}\;\sim \;N\left(0,\sigma_{\delta \rho }^{2}\right)$ and $\eta_{RR}\;\sim \;N\left(0,\;\sigma_{\delta \dot{\rho }}^{2}\right)$. The variance is related to the discriminators used for the measurement residuals, which are modeled as

(14) $$\sigma_{\delta \rho }^{2}=\frac{\lambda_{CA}^{2}}{2{\left(T\frac{C}{N_{0}}\right)}^{2}}+\frac{\lambda_{CA}^{2}}{4T\frac{C}{N_{0}}}$$

(15) $$\sigma_{\delta \dot{\rho }}^{2}={\left(\frac{\lambda_{L1}}{\pi T}\right)}^{2}\left(\frac{2}{{\left(T\frac{C}{N_{0}}\right)}^{2}}+\frac{2}{T\frac{C}{N_{0}}}\right).$$

Since the integration period and wavelengths are constant, the carrier-to-noise density ratio, $C/N_{0}$, is what dictates the measurement quality. The measurement covariance matrix is a diagonal matrix where the elements are Equations (14) and (15) with the appropriate estimates of $C/N_{0}$ applied.

#### 2.1.4. Vector Tracking NCO Updates

Once the measurements have been applied to the filter, the NCOs are updated using

(16) $$f_{code}^{k+1}=\frac{f_{CA}T-{\hat{\theta }}_{k}}{\left(t_{t}^{k+1}+\frac{{\hat{\rho }}_{k+1}}{c}\right)-t_{r}^{k}}$$

and

(17) $$f_{carrier}^{k+1}=-\frac{{\hat{\dot{\rho }}}_{k+1}}{\lambda_{L1}}.$$

In Equation (16), the code frequency for the next accumulation period, $f_{code}^{k+1}$, is a function of the nominal code frequency, $f_{CA}$, the integration period, the current code phase estimate, ${\hat{\theta }}_{k}$, the transmit time of the end of the next data bit, $t_{t}^{k+1}$, the estimated pseudorange at the next transmit time, ${\hat{\rho }}_{k+1}$, the speed of light, *c*, and the current receive time $t_{r}^{k}$. The code NCO is calculated by dividing the number of chips remaining in the data bit by the predicted change in the receive time across that interval. The carrier NCO in Equation (17) is updated by scaling the predicted pseudorange rate ${\hat{\dot{\rho }}}_{k+1}$ into a Doppler frequency using the wavelength.

#### 2.1.5. Overview of the Beamforming Algorithm

With the receiver architecture outlined, the beamforming techniques can be introduced. The principle of beamforming is that the feeds from the phased antenna array can be phase-shifted and summed such that signals of interest experience constructive interference or other sources experience destructive interference. To steer an amplifying beam towards the transmitter of the desired signal, either the DOA of the transmitter must be known in the antenna frame, or the array must use a “blind” adaptive method to find the correct weighting of antenna feeds.

For deterministic beam steering of received GNSS signals, the direction of arrival can either be determined from the navigation states or from the signal states. In both deterministic cases, however, the antenna array needs to be calibrated. Determining DOA from the navigation states requires knowledge of the satellite ephemeris, time, and the attitude of the platform. Attitude can be determined from additional sensors, but for ground vehicles, GPS course can provide sufficiently accurate attitude given the wide beams produced by arrays with limited numbers of elements. It should be noted that attitude can also be determined by a phased array antenna, but signal-based DOA would already be available in those cases. To determine DOA from the signal states, generally GNSS signals need to use a post-correlation technique. GNSS signals are received at powers below the noise floor, which makes direction finding from raw RF data difficult. Once the data from every antenna has been correlated, a hyper-resolution algorithm such as multiple signal classification (MUSIC) or carrier phase measurements can be used to determine the DOA of the correlated signal in the antenna frame [25]. However, DOA estimates from signal parameters suffer in low $C/N_{0}$ environments, and the presence of multipath in the correlations can bias the DOA estimates. Once the look direction to the transmitter is known, the proper phase shifts for each antenna feed can be calculated and applied to each channel.

For adaptive methods, calibration of the antenna is not required. Some sort of estimation or recursive filter can be used to find the weights that maximize the signal of interest’s strength. One of the traditional adaptive beamformers in GNSS is the eigenbeamformer, where optimal weights for beam steering are the eigenvector corresponding to the dominant eigenvalue of the autocorrelation matrix of a received signal [26]. This is the beam steering algorithm used in [21], but the eigenbeamformer can be degraded by an NLOS signal that would appear in the autocorrelation matrix. Another adaptive beam steering algorithm is a recursive least mean squares algorithm, where the summed signal is recursively adapted to align with a signal replica. This method generally requires phase lock, which is the weakest link of a GNSS receiver, but iterative adaptations with algorithms like space alternating generalized expectation maximization (SAGE) have been made to make it more robust [27]. Another algorithm worth noting is the minimum variance distortionless response (MVDR), which is a common fusion of deterministic and adaptive beamformers. This algorithm uses deterministic information about DOA to constrain a unity gain in that look direction while minimizing RFI in other directions.

Lastly, consideration needs to be made about how the phase shifts on the received signals need to be applied. For RFI scenarios where the interference will be common on every channel, the phase shifting can be applied directly to the RF feeds. However, for beam steering, it is often easier to apply the weights with every feed mixed to baseband, and some communication systems will apply the phase shift with the local oscillator directly [28]. However, for GNSS software radios the beamforming is generally performed digitally on the baseband samples. Typical GNSS antenna arrays are small to be available for a variety of platforms and will therefore have a limited number of spatial degrees of freedom. GNSS arrays will often have many more visible emitters than degrees of freedom, and the maximization of a single incoming signal requires the use of all degrees of freedom. However, by exploiting the correlation properties of GNSS signals, signals can be treated independently from one another. This allows for a unique set of weights to be applied to every emitter that can consume all degrees of freedom for amplification of that signal. To apply multiple weight sets simultaneously, the data must be processed digitally. So, for simultaneous maximization of all signals in view, a digital beamformer is required to apply unique weight sets for every emitter.

**Figure 2:**
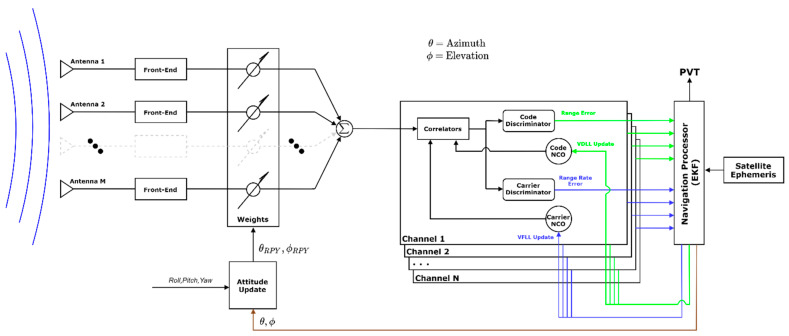
A diagram of the architecture of the beam steering vector tracking GNSS receiver.

The beamformer used in this work is a digital deterministic delay and sum beamformer that weights all incoming feeds to constructively interfere to amplify the signal. The weights applied are

(18) $$W_{m}=e^{-\frac{j2\pi \hat{r}d_{m}}{\lambda_{L1}}},$$

where the weight of the $m^{th}$ antenna element is calculated from the dot product of the element location, $d_{m}$, and the estimated look-direction to the source, $\hat{r}$, scaled by the wavelength of the signal. The look direction must be known in the antenna array’s frame. Satellite ephemeris provides the look direction in the navigation frame, which can be rotated into the body frame with an attitude estimate from the receiver. For this work, the mounting angle of the antenna is known, and the attitude of the vehicle is estimated using GNSS vehicle course. A diagram of the beam steering vector tracking receiver is shown in Figure 2. While multiple beamformers were presented, the deterministic navigation-based delay and sum beamformer works well with a vector tracking architecture applied to ground vehicles and is not subject to potential NLOS-caused distortions. A simple comparison of multiple beam steering algorithms using live sky data is presented in Section 4.

### 2.2. Simulation of Antenna Array Performance

**Figure 3:**
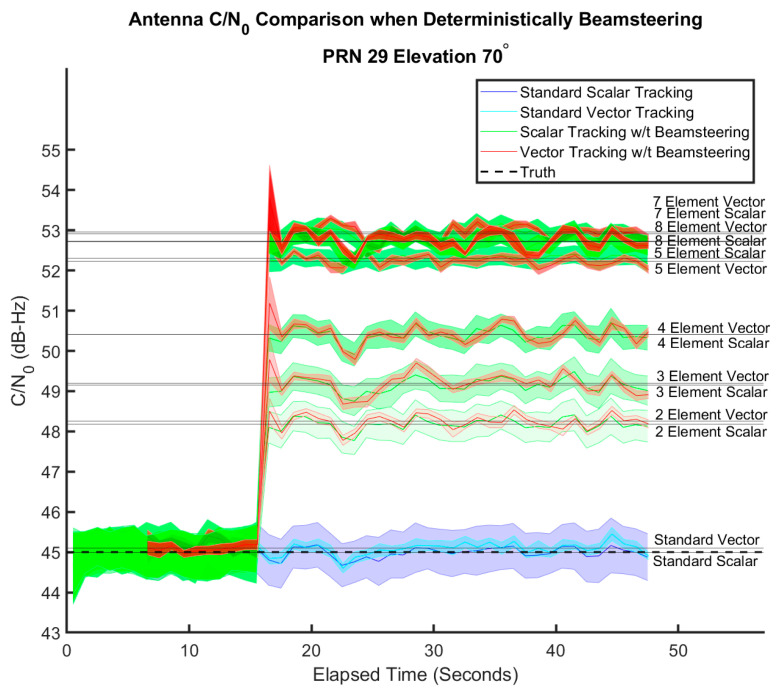
Comparison of $C/N_{0}$ for a vector and scalar receiver given a variety of array sizes.

This receiver design is not dependent on any CRPA layout. To show the potential amplification of the received signal, a series of arrays are tested in a signal level simulation for both a scalar and a vector tracking receiver to show the improvement in $C/N_{0}$ from the addition of beam steering. The arrays tested are a 2-element uniform linear array, a triangular 3-element array, a 4-element uniform rectangular array, a 5-element uniform circular array, a 7-element centered hexagonal array, and an 8-element uniform circular array. Each element is modeled as isotropic. The scalar receiver is using a second-order DLL/FLL design. The results are presented in Figure 3.

**Figure 4:**
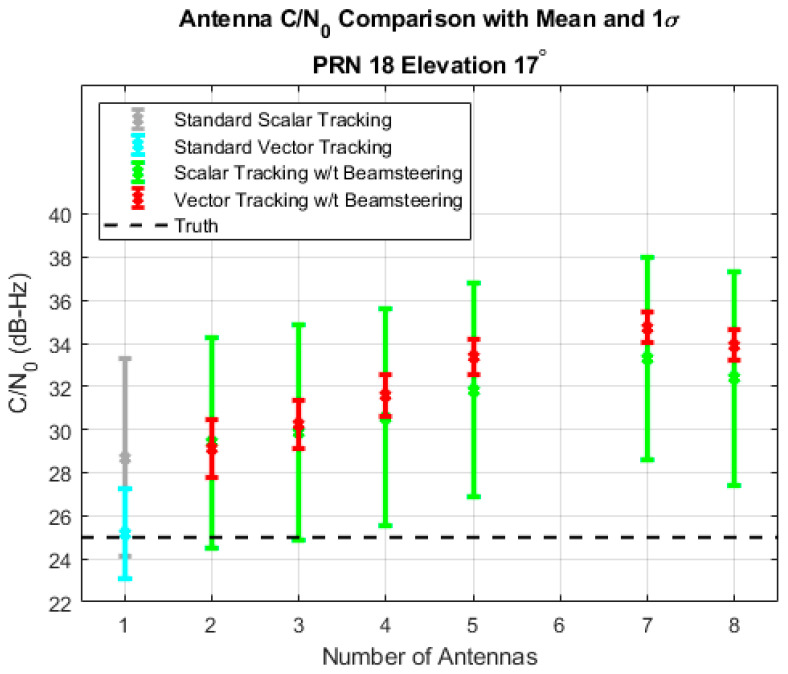
Comparison of $C/N_{0}$ for a vector and scalar receiver given a variety of array sizes and a true $C/N_{0}$ of 25 dB-Hz.

Figure 3 shows the $C/N_{0}$ of the two receivers for a given number of elements over time. The $C/N_{0}$ is averaged over every second of data, and the mean and one standard deviation are used to form the envelopes plotted above. At the 15 s mark, beam steering is enabled for each receiver. The arrays provide near the expected gain from constructive interference of summing a number of phase-aligned signals equal to the array size. There is no difference in amplification between the scalar and vector receivers, as expected. However, the vector design does allow for a decreased variance in the $C/N_{0}$ estimate over the scalar due to decreased variance in correlation amplitude because of the navigation state-based NCOs. Further benefits of the vector tracking will be shown in low power signal tracking in Figure 4.

For this simulation in Figure 4, all signals were initialized at 45 dB-Hz and then decreased to 25 dB-Hz after receivers enabled beam steering. The plot shows the average $C/N_{0}$ and one standard deviation over the low-power simulated data. While both receivers can maintain a lock on the signal, the vector tracking receivers are maintaining a much tighter variance on the $C/N_{0}$ estimate. In Figure 3, vector tracking was shown to keep a tighter variance, but the standard deviations were both sub-1 dB-Hz for all arrays. However, in the low signal power case, vector receivers can maintain a standard deviation below 2 dB-Hz for all arrays while the scalar receivers have standard deviations of approximately 5 dB-Hz and nearly 2 dB-Hz lower means for the beam steering arrays. Both receivers have identical $C/N_{0}$ estimators. The only difference comes from the quality of the correlations from the tracking loop. This experiment was also performed at 20 dB-Hz, and every scalar receiver failed to track while the vector receiver maintained a similar amplification over the nominal $C/N_{0}$ for all arrays.

## 3. Methods for Experimental Collection

**Figure 5:**
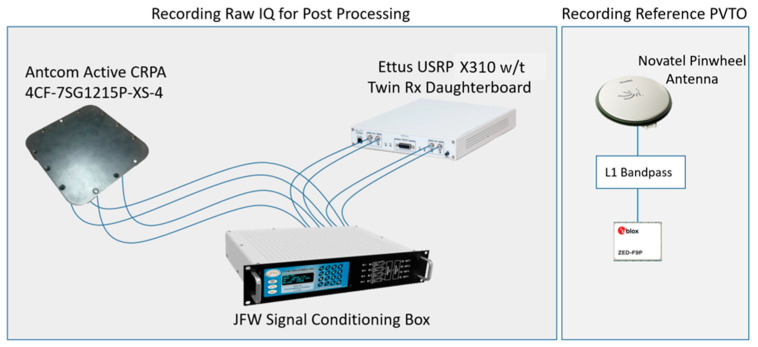
Hardware setup for dynamic live-sky data collection.

To test the presented receiver algorithms from Section 2, synchronized live-sky RF data needed to be collected using a CRPA array. Figure 5 shows an overview of the data collection setup. The CRPA antenna used for the collection was the Active CRPA 4CF-75G121SP-XS-4 (Antcom Corporation, Torrance, CA, USA). The CRPA is an active four-element right-hand circularly polarized (RHCP) antenna array that receives GPS L1 and L2 frequencies. The array is square with $0.4\;\lambda_{L1}$ element spacings. The front-end recorder was an USRP X310 (Ettus Research, Austin, TX, USA), integrated with a twin Rx daughterboard with a common local oscillator [29]. Each channel has up to 160 MHz bandwidth, but the sampling rate on all four channels was set to 25 MHz. The data were recorded as int16 IQ sample pairs. Despite the active antenna array, the low receive power of GNSS signals was still a concern. A custom signal conditioning box (JFW Industries, Inc., Indianapolis, IN, USA) was placed in-line between the USRP and the Antcom antenna. The signal conditioning box provided programmable bias tees that powered the CRPA and low noise amplifiers (LNA) for received signal amplification. The Ettus USRP X310 offers programmable gains like that of the JFW signal conditioning box. To prevent clipping of the recorded signal, an automatic gain controller (AGC) was programmed in UHD to continuously adapt the USRP gains to maintain signal quality. The JFW gains were set to raise each signal stream to −2.5 dBFS before the USRP AGC could fine-tune the signal data to better quality.

Along with the USRP collecting four-channel IQ data, a Ublox F9 receiver (Ublox Holding AG, Thalwil, Switzerland) was used to record reference data for analysis [30]. The Ublox was connected to a Novatel 702 GG Pinwheel antenna (NovAtel Inc., Calgary, Alberta, Canada). A GPS L1 bandpass filter was placed in line to keep both the proposed receiver and the Ublox F9 limited to only L1 signals. The Ublox F9 has an accuracy of 1.5 m in the horizontal and 2.25 m in the vertical direction [30]. A diagram of the Ublox hardware setup is also shown in Figure 5.

**Figure 6:**
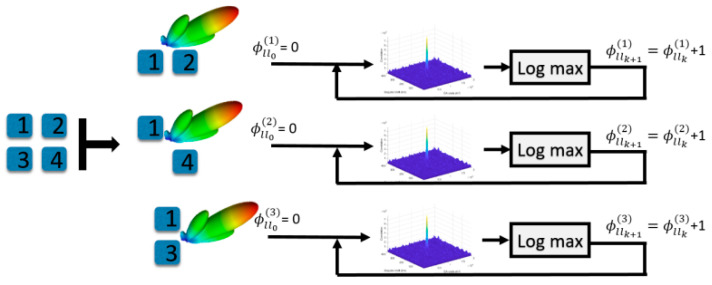
Overview of the in situ calibration process for determining beam steering phase shifts.

A calibration was necessary to account for phase offsets within the collection setup as well as the phase pattern of the antenna for beam steering to be possible. An in situ calibration was performed that would jointly capture the data collection hardware offsets as well as the antenna manifold. The 4-element array was digitally separated into a series of 2-element sub-arrays, each using a designated common lead antenna. Each sub-array would determine the expected delay to be applied to the auxiliary antenna feed based on known receiver and satellite states for constructive interference. Then, iterative scans of phase offsets in one-degree increments were used to determine the phase offset that would maximize the correlation of the sub-array. With the delay for every sub-array determined, all delays can be used to sum the incoming signal into a single feed that is fed to the VTL for maximum constructive interference of the signal. A diagram of the in situ calibration sub-arrays is shown in Figure 6. This scanning process had to be performed for every desired satellite in view. Due to the wide beams provided by a deterministic 4-element array beam steering and the slow change in unit vectors between GNSS satellites and ground vehicles, the in situ calibration would provide accurate phase delays for producing the correct amplification until changes in the user attitude occurred. The user attitude was determined by GNSS vehicle course, and the scan would be repeated to handle the new system attitude states. While this in situ calibration process was successfully used to enable beam steering towards GNSS satellites, the process was computationally intensive, and using a calibrated antenna with a known manifold is recommended.

**Figure 7:**
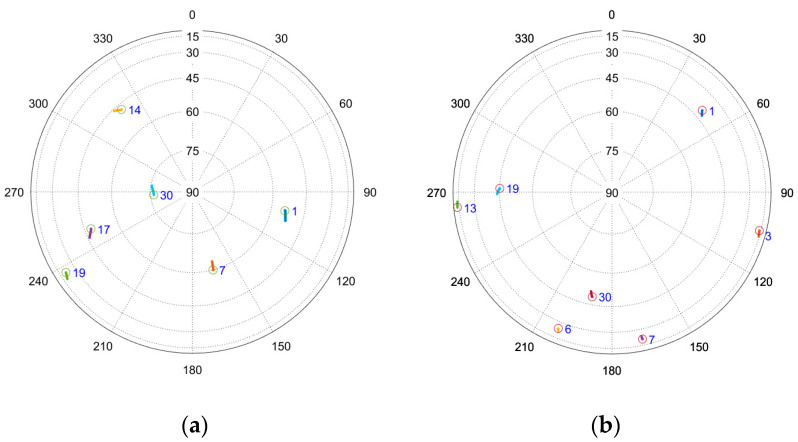
Satellite geometry for (**a**) the commuter route and (**b**) the campus route.

For the dynamic data collection, two routes were driven through Auburn, Alabama. The first route, called the commuter route, travels through a suburban and highway environment. The second route, called the campus route, travels through Auburn University, passing a series of dormitories, parking garages, and the football stadium. The commuter route features strong signal conditions and minimal multipath, while the campus route will feature occlusions and multipath for degraded signal conditions. The satellite geometries at the time of collection of both data sets are shown in Figure 7.

## 4. Results

The following sections will present results from experimental data collections. First, a simple comparison of beam steering algorithms will be shown, and dynamic results will be presented from along two routes: the commuter and the campus route.

### 4.1. Beam Steering Comparison

**Figure 8:**
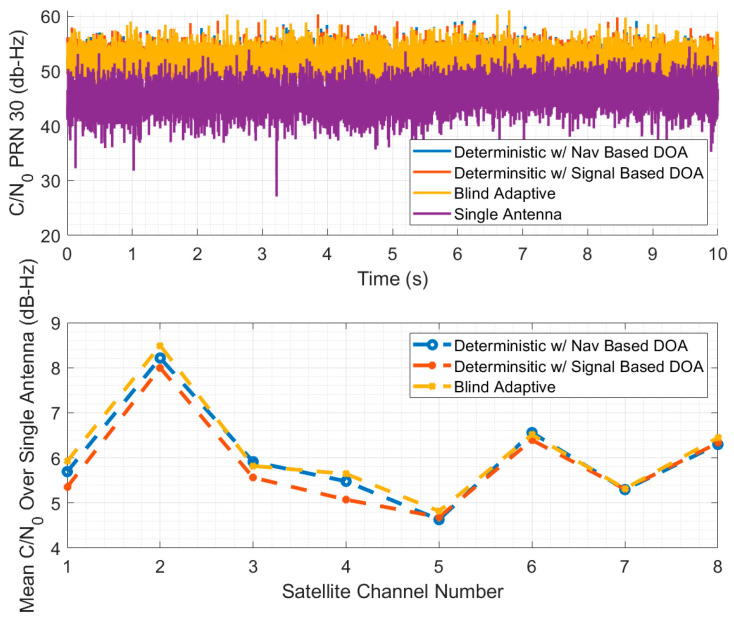
Comparison of $C/N_{0}$ estimates for different beam steering algorithms.

For the comparison of beam steering algorithms, three algorithms are used. The first algorithm is a deterministic delay and sum beamformer that estimates DOA from the navigation states. The second algorithm is a deterministic delay and sum beamformer that estimates DOA from the signal states using a MUSIC algorithm as shown in [25]. The third is an adaptive eigenbeamformer as presented in [26]. These data were collected while driving straight in a parking lot on the campus of Auburn University. The GNSS course was used as the attitude estimate for the navigation-based DOA beamformer. All beamformers used an identical scalar tracking loop to process the signals. The results are shown in Figure 8.

In the top plot of Figure 8, the estimated $C/N_{0}$ is shown for all three beam steering receivers and for a single antenna receiver for a single satellite as a function of time. The bottom part of the figure shows the mean improvement in $C/N_{0}$ over the single antenna receiver for all satellites in view. The satellite PRN 30 that is shown above is channel 8 in the lower plot. With the four-element antenna array, the expected gain is approximately 6 dB-Hz, and this improvement is roughly seen for every satellite for every beam steering receiver. While the blind adaptive receiver can quickly estimate the optimal gains, it only outperforms the deterministic methods by tenths of a dB-Hz. This result provides validation for the choice of the deterministic navigation-based beam steering algorithm for providing robust navigation with a simple beamforming implementation that is free from potential NLOS disruptions.

### 4.2. Commuter Route

**Figure 9:**
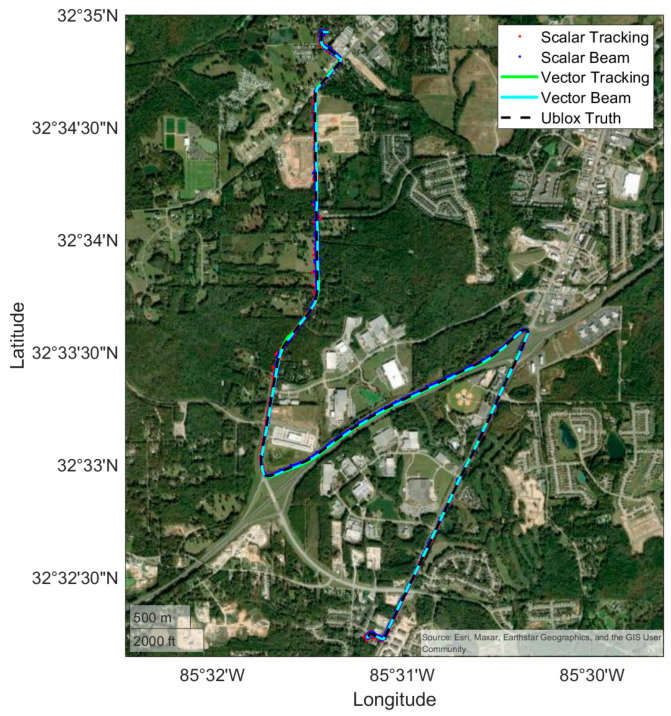
The positioning results from the commuter route.

**Figure 10:**
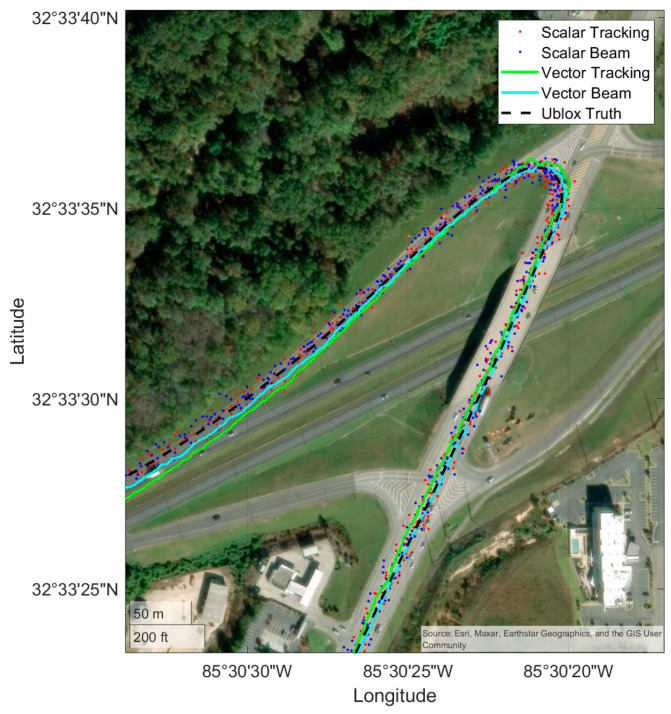
A zoomed-in view of positioning results through a corner of the commuter route.

**Figure 11:**
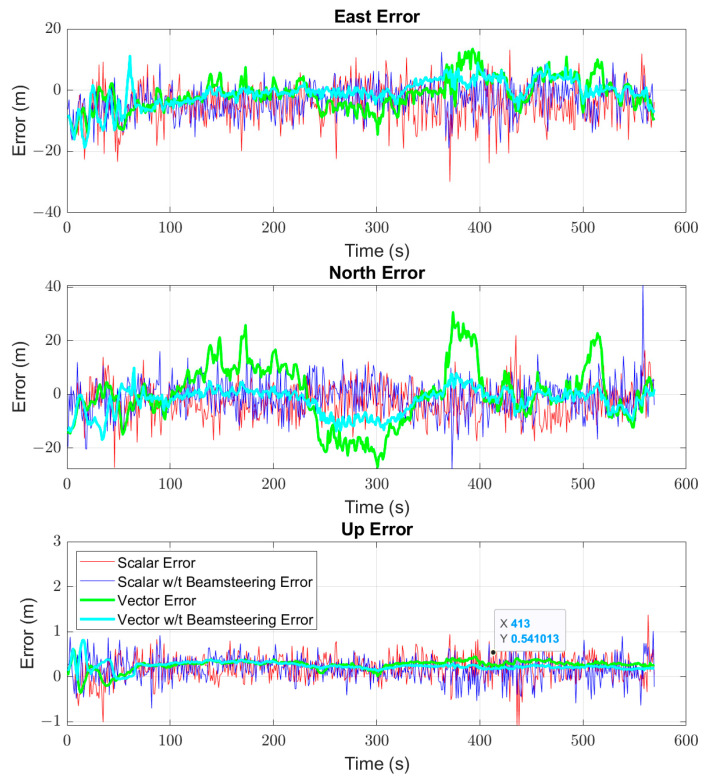
ENU positions for scalar and vector receivers both with and without beamforming relative to the Ublox position estimates as a function of time on the commuter route.

The first results displayed are for the commuter route. Positioning results are shown for a scalar and vector receiver, both with and without beam steering, as well as the Ublox. First, the full route is shown in Figure 9, and then a subsection of the route to highlight performance in open conditions is shown in Figure 10. The purpose of Figure 9 and Figure 10 is to provide an overview of the route taken and to provide a qualitative visualization of receiver performance relative to the environment. Figure 11 shows the quantitative results of the receiver performance relative to the Ublox receiver which is treated as a “truth” source. The vehicle varied its speed in the range of 10 to 35 m/s during the run as it covered both highways and residential roadways.

With multiple high-elevation satellites and obstructions limited to foliage on the roadside, all receivers performed well as expected. This route provides a baseline for the relative performance of the receivers to the Ublox in reasonable everyday signal conditions but does not yet highlight the benefit of vector tracking or antenna arrays.

Figure 11 shows the east, north, and up (ENU) position results relative to the Ublox receiver, while Table 1 contains the mean and standard deviation of the receivers relative to the Ublox. The addition of beam steering, even in a good environment, resulted in improvement in positioning for both the scalar and vector receivers. The most noticeable improvement is in the decrease in variance for the vector receiver, particularly in the north direction. This can be seen in Figure 11 as the sustained errors for the single antenna VTL in the 250–300 s and 375–400 s in the north direction are removed with the addition of beam steering. Otherwise the solutions were comparable in clear sky environments.

**Figure 12:**
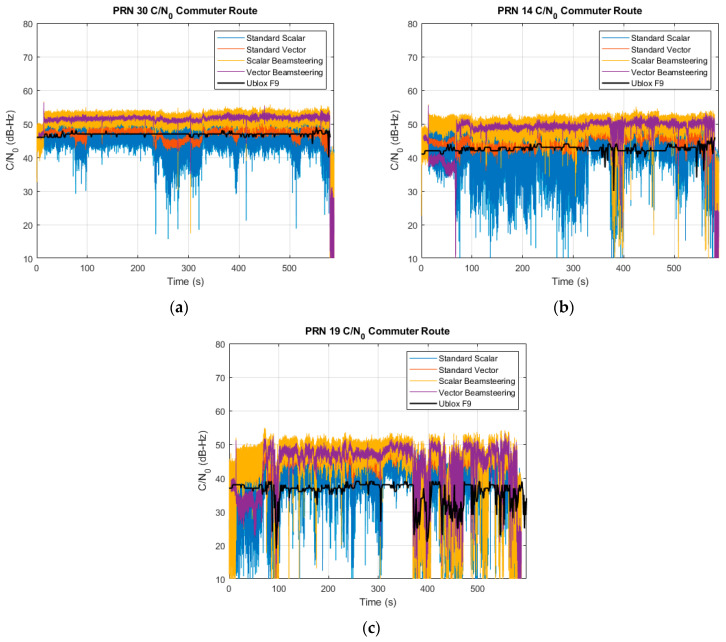
Estimated $C/N_{0}$ for all receivers for a (**a**) high elevation, (**b**) middle elevation, and (**c**) low elevation satellite on the commuter route.

While the positioning results were comparable due to the overall strong signal environment on the commuter route, the vector tracking and beam steering tend to show the most benefit in the tracking of low-power signals. The $C/N_{0}$ of three satellites of high, mid, and low elevation is shown in Figure 12. The elevations for PRNs 30, 14, and 19 are approximately 70, 50, and 20 degrees, respectively. The improvement of estimated $C/N_{0}$ from beam steering is readily apparent at all elevations. There is an expected 6 dB of gain from 4-element beam steering, and in all conditions, both scalar and vector receivers see the expected amplification. Of note is the performance of the beam steering vector tracking receiver for the low elevation satellite. It maintains a higher estimate of $C/N_{0}$ than the Ublox and a tighter variance than the scalar receiver despite the degradation of the incoming signal.

### 4.3. Campus Route

**Figure 13:**
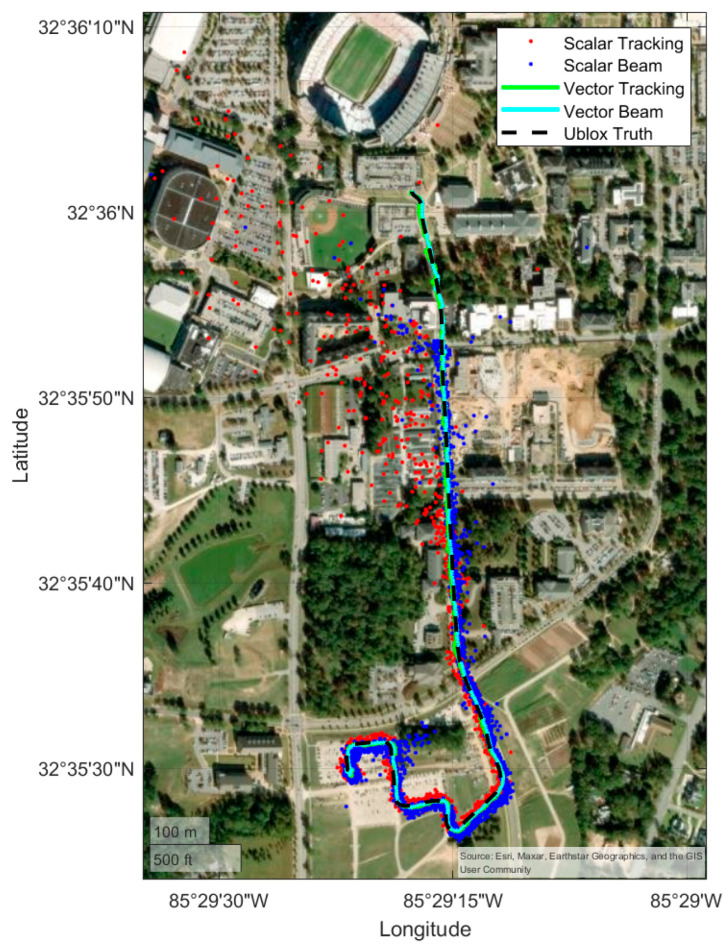
Positioning results from the campus route.

**Figure 14:**
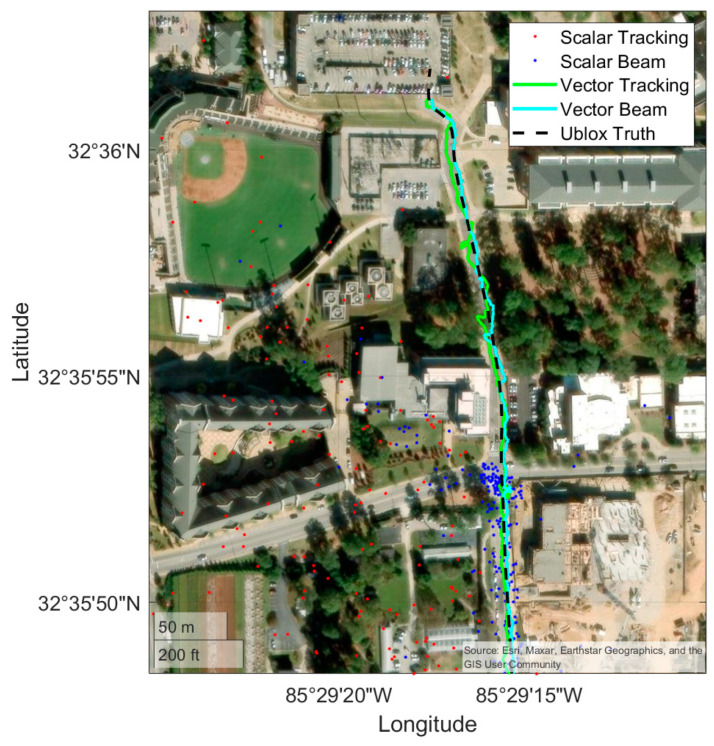
A zoomed-in view of the campus route showing where the scalar receivers began to fail.

**Figure 15:**
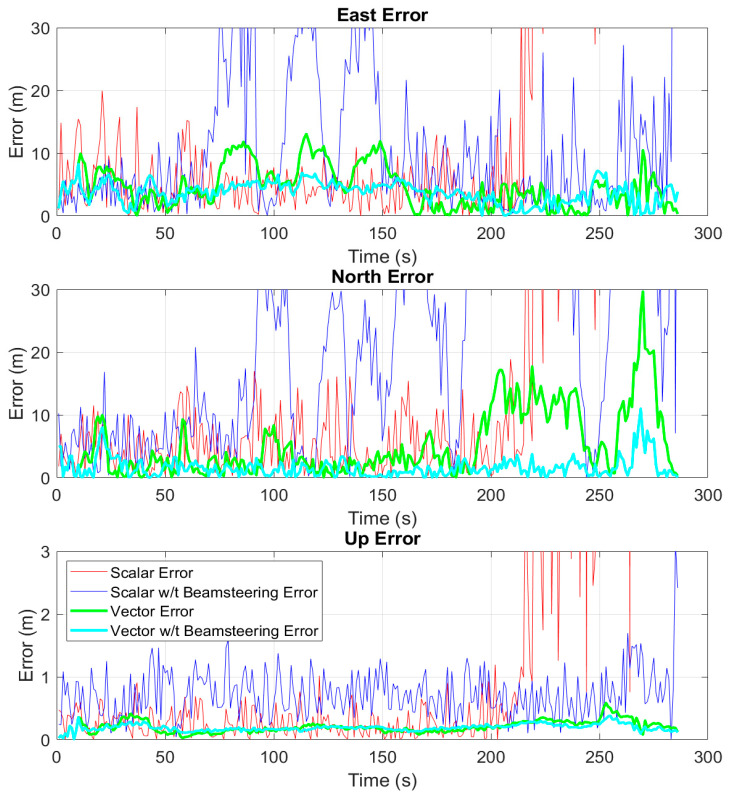
ENU positions for scalar and vector receivers both with and without beamforming relative to the Ublox position estimates as a function of time on the campus route.

The following results are for the campus route. Figure 13 shows the positioning results of the full campus route, and Figure 14 shows a subsection at the end of the campus route where the scalar receivers position solutions begin to drift. Again, Figure 13 and Figure 14 provide a qualitative overview of the route and solution performance relative to the course driven, while Figure 15 provides the quantitative comparison. The campus route begins at the south end of the campus of Auburn University and drives northward into the heart of campus. As the vehicle moves north, the line-of-sight to the satellites begins to be occluded by some heavy foliage, and there are opportunities for multipath while driving by academic buildings, parking garages, and the football stadium. Most noticeable from Figure 14 is that the scalar receivers both begin to fail. The beam steering scalar loop can keep up with the true PVT for longer, but it too begins to bias the PVT estimate. The vector receivers both stay accurate to the truth route for the duration of the run.

The position results from the four receivers relative to the Ublox are presented in Table 2. Here, the benefit of the vector tracking can be seen readily over the scalar tracking. In a degraded environment with multipath, isolated scalar loops can fail to track, while vector loops aid the degraded channels and keep accurate estimates of signal parameters. Furthermore, the improvements from adding beam steering can be seen as well. The scalar receiver can hold on to its channels for longer with beam steering than it can with a single antenna, and the beam steering vector receiver is able to provide a more accurate position solution. Figure 15 shows the ENU PVT errors relative to the Ublox as a function of time. Again, the vector receivers can keep a consistent position fix despite degrading conditions. The addition of beam steering to the vector receiver also provides more consistency to the navigation solution. From 200 to 250 s in the north direction, beam steering provides a nearly 10 m improvement over the single antenna counterpart. Similarly, in the east direction, the addition of beam steering removes 5 m of error from the 75 to 150 s mark.

**Figure 16:**
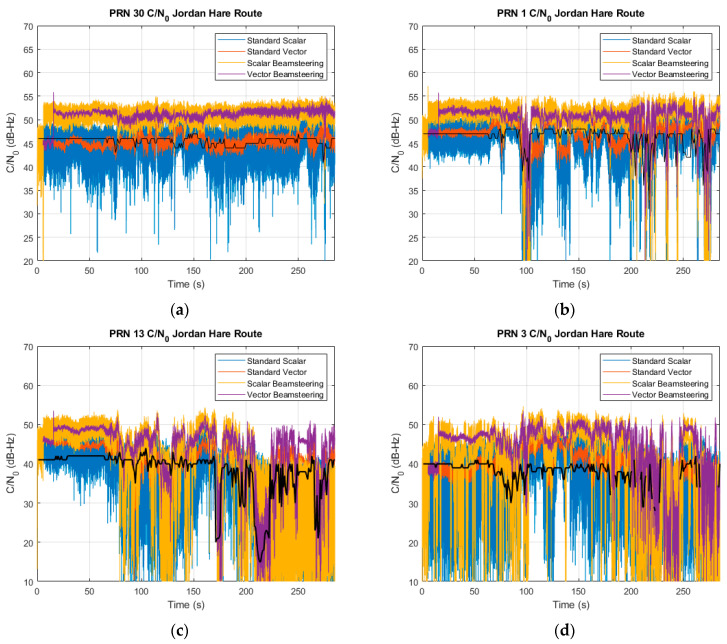
$C/N_{0}$ estimates of a (**a**) high elevation, (**b**) middle elevation, (**c**) low elevation, and (**d**) degraded low elevation satellite along the campus route.

The full benefit of beam steering in conjunction with vector tracking can be better seen in the individual signals’ received power. Figure 16 shows the $C/N_{0}$ of four received signals for all the receivers. PRNs 30, 1, 13, and 3 are shown in order of decreasing angles of elevation of approximately 50, 40, 15, and 12 degrees, respectively. In clean line-of-sight scenarios like PRN 30, using beam steering resulted in roughly a 6 dB improvement for both scalar and vector receivers, but the vector architecture enables stability in correlation magnitude that in turn decreases the variance in $C/N_{0}$ estimation. On channels with some blockages or multipath, like PRN 1, the beam steering allows for quality measurements despite short-term degradations. The vector beam steering receiver tracks closely with the Ublox in the magnitude and duration of the $C/N_{0}$ dips but consistently maintains its array-provided power advantage. On PRN 13, the scalar receiver with beam steering is nearly unable to survive the degradation despite the signal amplification, but the vector with beam steering receiver can track the signal through the low power duration and quickly regain the power advantage over the Ublox when signal conditions improve afterwards. On PRN 3, the Ublox receiver is unable to track the signal for the full duration, dropping the signal from the 230–250 and 260–280 s marks. The vector beam steering receiver can maintain a usable measurement for the entire interval.

## 5. Conclusions

This work introduced algorithms for the combination of a vector tracking receiver and a controlled reception pattern array in a software-defined radio. These algorithms were then experimentally verified with dynamic live-sky data in both clear and multipath-rich environments. The experiments successfully demonstrated that the combination of robust receiver algorithms in combination with phased arrays can provide superior navigation performance over traditional single antenna receivers. While CRPAs primary use case is in the mitigation of radio interference, their ability to beam steer can increase received signal power, which in turn improves signal parameter estimates, receiver measurements, and receiver navigation. This was demonstrated by the beam steering receiver’s ability to achieve the expected 6 dB in signal power amplification over single antenna receivers as well as the vector tracking receiver’s ability to decrease position errors by 5 to 10 m in the north and east directions by including beam steering. The combination of beam steering and vector tracking was also capable of providing similar tracking performance as that of the Ublox receiver while also maintaining a higher signal power estimate and surviving degradations that caused the Ublox to temporarily lose signal lock.

Future considerations should examine the inclusion of an INS system for a deeply coupled receiver that can be used to further improve signal detection thresholds and degraded signal navigation as well as investigate multi-frequency receivers for the use of more multipath-resistant signals as well as pilot channels for optimal integration periods.

## Figures and Tables

**Table 1:** Commuter route ENU position error statistics relative to the Ublox F9.

ENU Errors: Mean and Standard Deviation (m)	$\mu_{E}\pm \sigma_{E}$	$\mu_{N}\pm \sigma_{N}$	$\mu_{U}\pm \sigma_{U}$
Scalar	5.99 ± 4.73	5.55 ± 4.13	0.29 ± 0.22
Scalar Beam Steering	4.88 ± 3.60	5.26 ± 4.21	0.26 ± 0.20
Vector	4.37 ± 3.24	8.69 ± 7.04	0.27 ± 0.08
Vector Beam Steering	3.18 ± 3.07	3.38 ± 3.75	0.25 ± 0.09

**Table 2:** Campus route ENU position error statistics relative to the Ublox F9.

ENU Errors: Mean and Standard Deviation (m)	$\mu_{E}\pm \sigma_{E}$	$\mu_{N}\pm \sigma_{N}$	$\mu_{U}\pm \sigma_{U}$
Scalar	367.51 ± 1865.48	253.49 ± 1315.30	15.57 ± 80.56
Scalar Beam Steering	11.40 ± 16.41	25.13 ± 22.49	0.73 ± 0.384
Vector	4.67 ± 3.26	5.25 ± 5.30	0.20 ± 0.10
Vector Beam Steering	3.70 ± 1.69	1.63 ± 1.59	0.19 ± 0.05

## Data Availability

Data available on request from the authors.
